# Coinfection Patterns of *Mycoplasma pneumoniae* with Other Respiratory Pathogens in Children

**DOI:** 10.3390/ijms27114925

**Published:** 2026-05-29

**Authors:** Elena-Roxana Matache (Vasilache), Gabriela Gurau, Nicoleta-Maricica Maftei, Andreea Eliza Zaharia, Manuela Ciocoiu, Madalina Nicoleta Matei, Aurel Nechita, Lucian-Daniel Peptine, Diana-Andreea Ciortea, Dana Tutunaru

**Affiliations:** 1 Faculty of Medicine and Pharmacy, Research Center in the Medical-Pharmaceutical Field, “Dunarea de Jos” University of Galati, 800008 Galati, Romania; elena.matache@ugal.ro (E.-R.M.); zaharia.eliza03@gmail.com (A.E.Z.); madalina.matei@ugal.ro (M.N.M.); aurel.nechita@ugal.ro (A.N.); lucian.peptine@ugal.ro (L.-D.P.); diana.ciortea@ugal.ro (D.-A.C.); dana.tutunaru@ugal.ro (D.T.); 2 “Sf. Ioan” Emergency Clinical Hospital for Children, 800487 Galati, Romania; 3 Department of Morpho-Functional Sciences II, “Grigore T. Popa” University of Medicine and Pharmacy, 700115 Iasi, Romania; manuela.ciocoiu@umfiasi.ro; 4 Medical Laboratory Department, “Sf Apostol Andrei” Emergency Clinical Hospital, 800578 Galati, Romania

**Keywords:** *Mycoplasma pneumoniae*, pneumonia, coinfection, children, respiratory viruses, respiratory bacteria

## Abstract

*Mycoplasma pneumoniae* (MP) is a frequent cause of pediatric pneumonia, but the clinical relevance of respiratory coinfections remains incompletely defined. We conducted a retrospective cohort study of 192 hospitalized children with multiplex RT-PCR-confirmed MP infection between April 2023 and November 2025. Patients were classified as MP monoinfection (123/192, 64.1%), MP with viral coinfection (34/192, 17.7%), or MP with bacterial/mixed coinfection (35/192, 18.2%) and children with coinfection were younger than those with MP infection alone, with median ages of 6.5 years (IQR 1–11) in the viral coinfection group and 5 years (IQR 1–7) in the bacterial/mixed coinfection group, compared with 11 years (IQR 7–14) in children with MP alone (*p* < 0.001). Respiratory failure occurred in 36% of children with MP infection alone, 65% of those with viral coinfection, and 31% of those with bacterial/mixed coinfection. In adjusted logistic regression, viral coinfection was independently associated with higher odds of respiratory failure relative to MP infection alone (aOR 3.37, 95% CI 1.49–7.94; *p* = 0.004), whereas bacterial/mixed coinfection was not (aOR 0.85, 95% CI 0.35–2.03; *p* = 0.725). In multinomial regression, increasing age was inversely associated with both viral coinfection (RRR 0.86, 95% CI 0.79–0.94; *p* < 0.001) and bacterial/mixed coinfection (RRR 0.79, 95% CI 0.72–0.87; *p* < 0.001). In negative binomial regression, evidence for longer hospitalization in the viral coinfection group was weaker and imprecise (IRR 1.16, 95% CI 0.99–1.37; *p* = 0.069). Fibrinogen differed across etiologic groups, with the lowest values in bacterial/mixed coinfection (*p* = 0.004). In this hospitalized pediatric cohort, MP coinfection was more frequent in younger children, and viral co-detection showed the clearest association with respiratory failure. These findings should be interpreted in the context of the retrospective design, modest subgroup sizes, and the limitations of upper-airway multiplex PCR co-detection.

## 1. Introduction

*Mycoplasma pneumoniae* (MP), an important atypical pathogen in respiratory infections, is responsible for 10–30% of community-acquired pneumonia (CAP) cases in hospitalized children worldwide [1].

After the SARS-CoV-2 pandemic, during which the number of cases of MP infections was significantly reduced, recent studies from different areas around the world indicated a resurgence of cases of MP pneumonia during 2023–2024 [2]. The Centers for Disease Control and Prevention (CDC) stated that August 2024 was the peak period for these infections. The increase was significant among children aged 5–17 years, rising from 3.5% to 7.4%, with a notable upward trend observed in children aged 2–4 years, with rates from 1% to 7.2% [3].

The coinfection prevalence involving children with MP and other pathogens published within studies have ranged from 30% to 60% [4,5]. According to some analyses, MPP coinfected with other pathogens increases the risk of complications [6]. Among severe CAP cases, 1–2% were found to be caused by coinfections with MP and other pathogens [7]. Over the past few years, cases of severe pneumonia coinfection with MP and viruses (rhinovirus, respiratory syncytial virus, adenovirus, parainfluenza, influenza virus, coronavirus and enterovirus) or bacteria (*S. pneumoniae*, *P. aeruginosa* and *S. aureus*) have been reported [8]. Moreover, it has been shown that these mixed coinfecting pathogens can cause consolidation and lung abscesses, leading to poor efficacy of antimicrobial therapy in acute phase. Complications such as pleural effusion, necrotizing pneumonia, and even an increased risk of mortality may occur [9,10,11,12,13].

However, the influence of coinfection with other respiratory pathogens over the clinical course of pneumonia with MP has been insufficiently investigated so far, leading to inconclusive results [14,15,16].

Therefore, we aimed to analyze the etiology of coinfection patterns in children hospitalized with *M. pneumoniae* to compare clinical and paraclinical profiles, and used multivariate models to evaluate length of stay, respiratory failure and predictors of coinfection status.

## 2. Results

**Figure 1:**
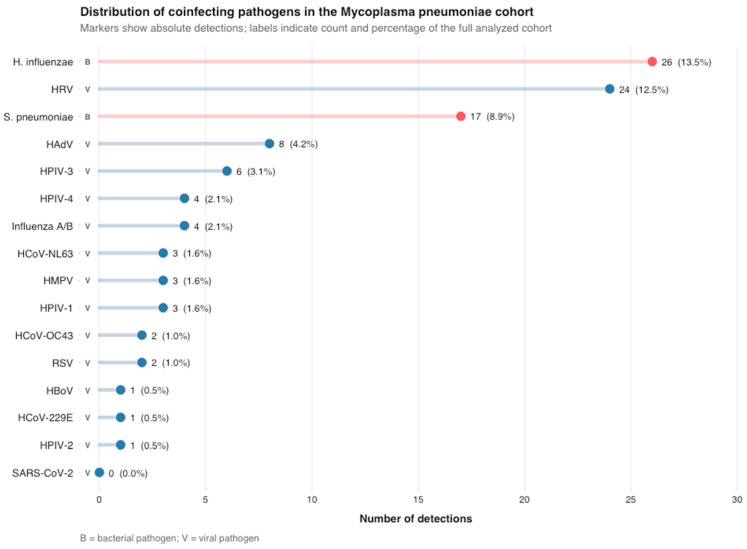
Distribution of coinfecting pathogens in the *M. pneumoniae* cohort. Bars indicate the total number of detections for each copathogen. *Haemophilus influenzae* and human rhinovirus were the most frequently detected organisms, followed by *Streptococcus pneumoniae*, whereas several viral pathogens were detected only sporadically.

A total of 192 hospitalized children with PCR-confirmed *Mycoplasma pneumoniae* (MP) infection were included in the final analysis. Of these, 123/192 (64.1%) had MP monoinfection, while 69/192 (35.9%) had coinfections detected by multiplex RT-PCR, including 34/192 (17.7%) with MP + viral coinfection and 35/192 (18.2%) with MP + bacterial/mixed coinfection. The overall median age was 9 years (IQR 5–13), and the median length of stay was 7 days (IQR 5–8). No deaths occurred during hospitalization. Recorded complications included respiratory failure, pleural effusion, pulmonary consolidation, ICU admission, and oxygen therapy requirement. These outcomes were analyzed descriptively across etiologic groups, whereas respiratory failure and length of stay were further evaluated using adjusted regression models. The distribution of non-MP pathogens detected in coinfected cases was heterogeneous, with *H. influenzae* and rhinovirus among the most frequent copathogens, followed by *S. pneumoniae*, while several other viruses were detected only sporadically (Figure 1).

Age structure differed markedly across etiologic groups. Children with MP monoinfection were older than those with coinfections, with median ages of 11 years (IQR 7–14) in the MP monoinfection group, 6.5 years (IQR 1–11) in the MP + virus group, and 5 years (IQR 1–7) in the MP + bacteria/mixed group (*p* < 0.001). This gradient was also evident categorically: the 7–17-year stratum accounted for 77% of MP monoinfections, compared with 50% of viral coinfections and 29% of bacterial/mixed coinfections, whereas infants aged <1 year represented only 1.6% of monoinfections, but 24% of viral coinfections and 17% of bacterial/mixed coinfections.

Clinical characteristics across the three etiologic groups are summarized in Table 1. Length of stay, ICU admission, and oxygen therapy were broadly comparable across groups at the descriptive level. Median hospitalization duration was 7 days (IQR 5–8) in MP monoinfections, 7 days (IQR 6–9) in MP + virus, and 6 days (IQR 4–9) in MP + bacteria/mixed infection (*p* = 0.2). ICU admission and oxygen therapy also showed no significant between-group differences (*p* = 0.8 for both), although respiratory failure was more frequent in the viral coinfection group.

Among presenting manifestations, rhinorrhea differed significantly across groups, being less frequent in MP monoinfection (6.5%) than in MP + virus (26%) and MP + bacteria/mixed infection (26%) (*p* < 0.001). By contrast, fever, dyspnea, vomiting, wheezing, pleural effusion, and pulmonary consolidation did not differ significantly between groups. Respiratory failure showed the clearest clinical separation: it occurred in 44/123 (36%) children with MP monoinfection, 22/34 (65%) with MP + virus, and 11/35 (31%) with MP + bacteria/mixed infection (*p* = 0.005). This identified the viral coinfection group as the subgroup with the highest crude respiratory burden.

Laboratory findings are summarized in Table 2. Several markers differed across etiologic groups. White blood cell count increased from MP monoinfection to the two coinfection groups, with median values of 8.61 × 10^9^/L, 10.38 × 10^9^/L, and 11.68 × 10^9^/L, respectively (*p* < 0.001). Lymphocyte count also differed significantly, rising from 1.77 × 10^3^/µL in monoinfection to 2.18 × 10^3^/µL in viral coinfection and 2.98 × 10^3^/µL in bacterial/mixed coinfection (*p* = 0.002). Hemoglobin and MCV were lower in the bacterial/mixed group than in monoinfection (12.37 vs. 13.10 g/dL, *p* = 0.002; 77.5 vs. 80.8 fL, *p* = 0.017). Median AST values were higher in both coinfection groups than in MP monoinfection (34 vs. 28 U/L, *p* = 0.002). CRP also differed between groups (*p* = 0.039), but without a consistent gradient, as the highest median values were observed in the monoinfection group and the lowest in the bacterial/mixed group. LDH did not differ significantly across groups (*p* = 0.15).

Coagulation markers are presented in Table 3. Fibrinogen showed the clearest etiologic gradient, with median values of 439 mg/dL in MP monoinfection, 372 mg/dL in MP + virus, and 284 mg/dL in MP + bacteria/mixed infection (*p* = 0.004). D-dimer values did not differ significantly between groups (*p* = 0.7). Thus, compared with MP monoinfection, the bacterial/mixed group showed substantially lower fibrinogen values, whereas no parallel difference was evident for D-dimer.

To distinguish descriptive from adjusted associations, three complementary multivariable models were fitted. In the logistic regression model for respiratory failure, viral coinfection remained independently associated with higher odds of respiratory failure compared with MP monoinfection (OR 3.37, 95% CI 1.49–7.94, *p* = 0.004), whereas MP + bacteria/mixed infection did not (OR 0.85, 95% CI 0.35–2.03, *p* = 0.725). Age was not independently associated with respiratory failure in this model (OR 1.00 per year, 95% CI 0.94–1.07, *p* = 0.981), nor were sex or residence (Table 4).

**Figure 2:**
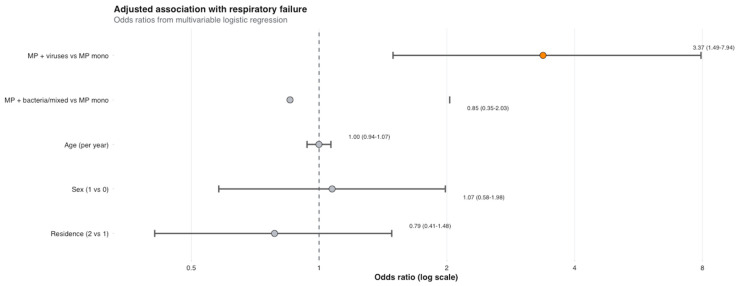
Forest plot of adjusted odds ratios for respiratory failure. Caption: Visualization of the adjusted estimates from the multivariable logistic regression model for respiratory failure. Points indicate adjusted odds ratios, and horizontal lines represent 95% confidence intervals on the logarithmic scale. The dashed vertical line denotes the null value (OR = 1). Viral coinfection showed the clearest positive adjusted association with respiratory failure relative to MP monoinfection. The orange point denotes the viral coinfection estimate, grey points denote the remaining model covariates, and the dashed vertical line indicates the null value of OR = 1.

**Figure 3:**
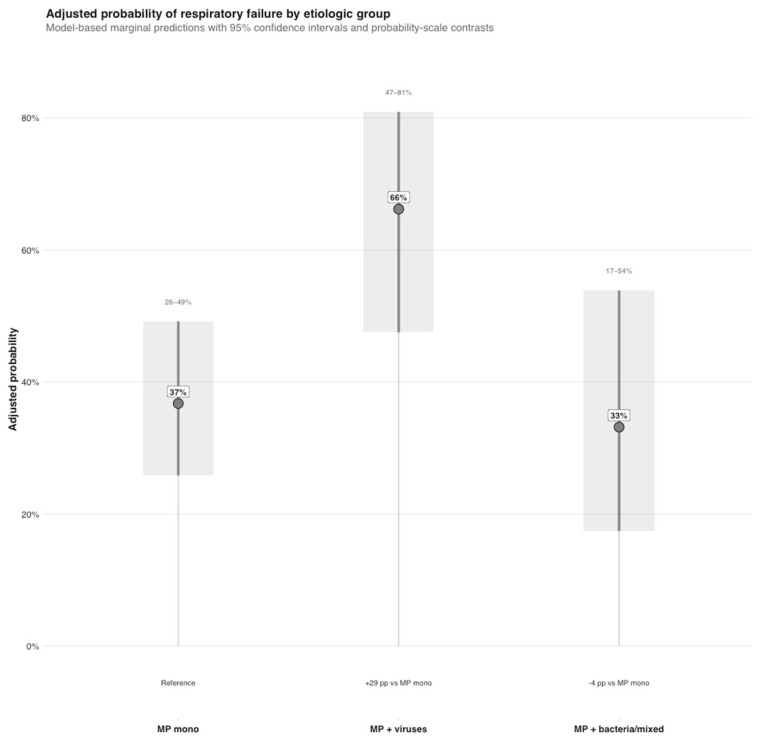
Adjusted predicted probability of respiratory failure by etiologic group. Caption: Marginal predicted probabilities derived from the multivariable logistic regression model for respiratory failure. Points indicate adjusted mean probabilities and error bars represent 95% confidence intervals. The viral coinfection group showed the highest adjusted probability of respiratory failure. The point estimates and error bars represent adjusted predicted probabilities and their 95% confidence intervals.

To facilitate interpretation of the logistic model, adjusted coefficients were also visualized graphically. The forest plot summarizes the direction and magnitude of each regression coefficient on the odds ratio scale, whereas the marginal prediction plot translates the same model into adjusted group-level probabilities of respiratory failure. Together, these figures distinguish coefficient-level inference from clinically interpretable absolute risk (Figure 2 and Figure 3).

Length of stay was analyzed using negative binomial regression. In the adjusted model, viral coinfection showed a borderline association with longer hospitalization relative to MP monoinfection (IRR 1.16, 95% CI 0.99–1.37, *p* = 0.069), whereas MP + bacteria/mixed infection did not (IRR 1.06, 95% CI 0.89–1.26, *p* = 0.525). Age showed a small positive association with length of stay (IRR 1.01 per year, 95% CI 1.00–1.03, *p* = 0.049). These results suggest a weaker etiologic signal for hospitalization duration than for respiratory failure (Table 5).

**Figure 4:**
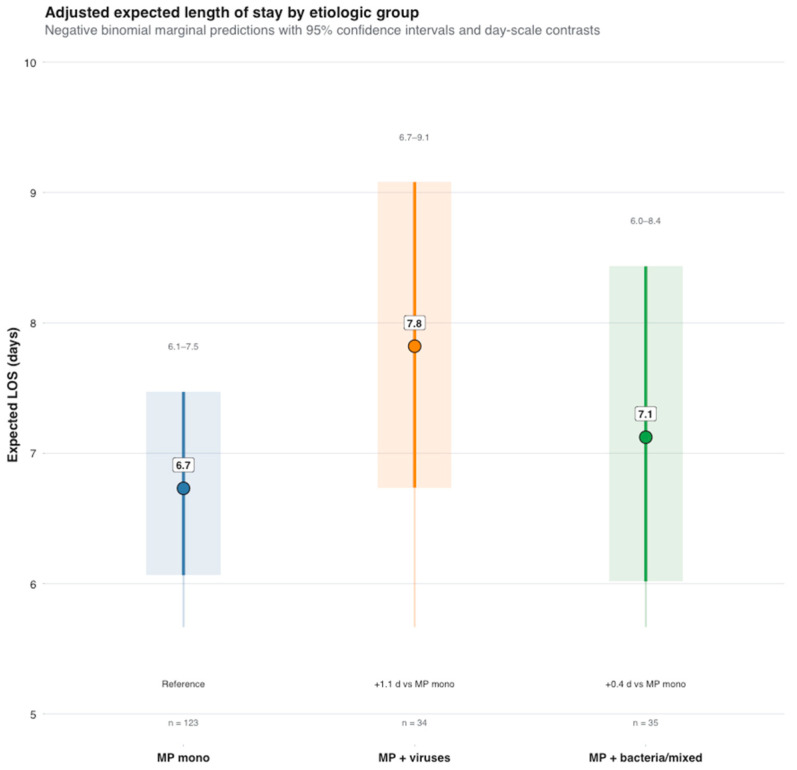
Adjusted expected length of stay by etiologic group. Caption: Model-based expected hospitalization duration estimated from the negative binomial regression model. Points indicate adjusted expected length of stay and error bars represent 95% confidence intervals. The figure is intended to aid clinical interpretation of the corresponding incidence rate ratios. Colors and shaded intervals are used only to visually distinguish etiologic groups and their 95% confidence intervals.

Because incidence rate ratios are less intuitive clinically than predicted group means, the negative binomial results were also displayed as adjusted expected length of stay by etiologic group. This visualization was used to assess whether the direction of the fitted LOS effect was consistent with the regression coefficients and whether the between-group differences were large enough to be clinically meaningful (Figure 4).

Predictors of etiologic group membership were evaluated by multinomial logistic regression, with MP monoinfection as the reference category. Age emerged as the strongest structural predictor of coinfection status. Each additional year of age was associated with lower relative risk of belonging to the MP + virus group (RRR 0.86, 95% CI 0.79–0.94, *p* < 0.001) and to the MP + bacteria/mixed group (RRR 0.79, 95% CI 0.72–0.87, *p* < 0.001). Sex and residence were not significantly associated with either coinfection category. These data indicate that coinfection in pediatric MP infection is strongly concentrated in younger children (Table 6).

**Figure 5:**
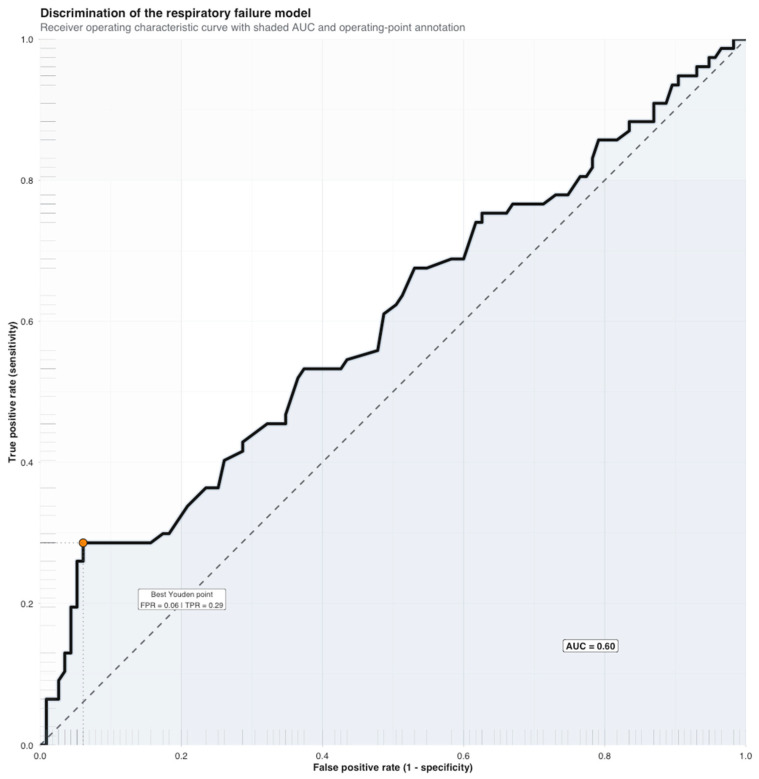
ROC curve for respiratory failure model. Caption: Receiver operating characteristic analysis of the multivariable logistic regression model for respiratory failure. The solid black curve summarizes model discrimination across classification thresholds, the shaded area represents the area under the curve, and the diagonal dashed line indicates no discrimination performance. This figure is presented as a model-performance summary rather than as primary etiologic evidence.

Model-based figures supported these findings visually. The forest plot highlighted viral coinfection as the only etiologic category with a clearly elevated adjusted odds ratio for respiratory failure, and the marginal probability plot showed the same pattern on the probability scale. Discrimination of the respiratory failure model was evaluated separately by receiver operating characteristic analysis. The resulting curve showed only modest discrimination (AUC = 0.60), indicating that etiologic grouping contributes to, but does not fully explain, respiratory failure risk. This figure is presented as a model-performance summary rather than as primary etiologic evidence (Figure 5).

**Figure 6:**
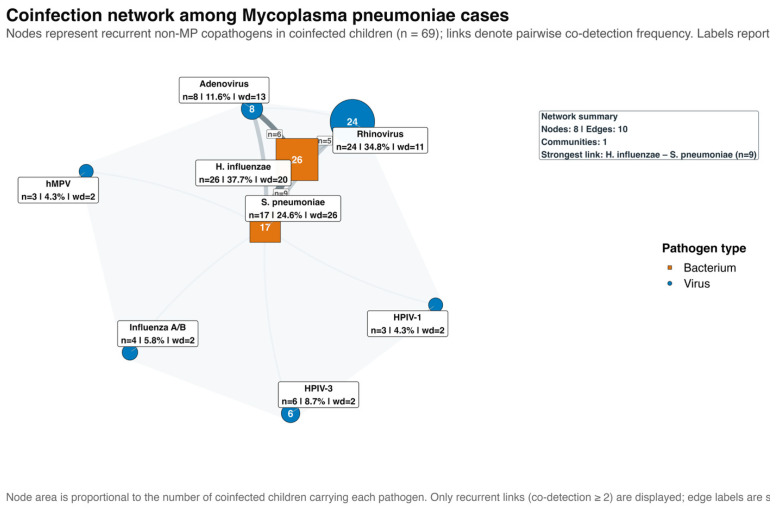
Coinfection network visualization. Caption: Exploratory network representation of recurrent non-MP copathogen co-detection patterns within the cohort. Nodes represent pathogens and edges represent observed co-detection links. The network is intended as a descriptive ecological map of coinfection architecture and should not be interpreted causally. Node color indicates pathogen type, node size reflects the number of coinfected children carrying each pathogen, and edge thickness reflects the frequency of observed co-detection links.

**Figure 7:**
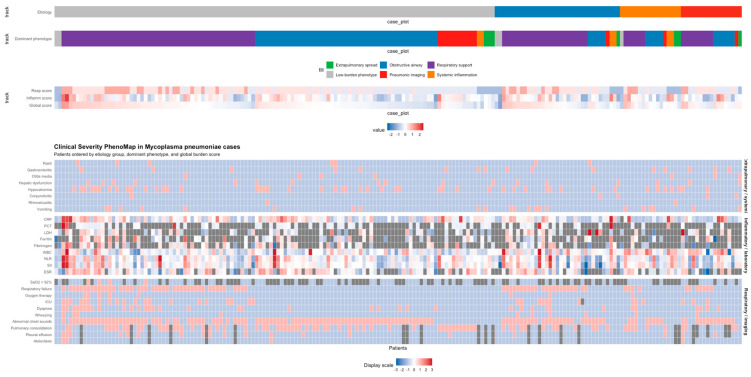
Clinical severity phenomap. Caption: Integrated heatmap–style phenotypic visualization of individual patients across etiologic grouping, selected clinical features, laboratory dimensions, and severity–related domains. This figure illustrates within-group heterogeneity and overlap between respiratory, inflammatory, imaging, and extrapulmonary patterns. It is presented as an exploratory integrative visualization rather than as primary inferential evidence. Colors represent relative feature values or categorical status within the heatmap and are used to display within–cohort phenotypic patterns rather than to define statistical significance.

Exploratory visualizations were used to complement, not replace, the regression analyses. The network plot summarizes recurrent copathogen co-detection patterns across the cohort, whereas the phenomap displays the distribution of respiratory, inflammatory, imaging, and extrapulmonary features across individual patients. Both figures are descriptive and hypothesis-generating. They are presented to illustrate within-group heterogeneity and patterns of co-detection, not to provide primary inferential evidence (Figure 6 and Figure 7).

Taken together, the results support three main conclusions: first, coinfection in pediatric MP pneumonia is strongly age-structured; second, viral coinfection, but not bacterial/mixed coinfection, is independently associated with respiratory failure; and third, laboratory and exploratory visual analyses suggest that etiologic groups capture distinct biological and phenotypic profiles rather than a single uniform severity continuum.

## 3. Discussion

In this cohort of 192 hospitalized children with PCR-confirmed *Mycoplasma pneu-moniae* infection, three findings define the overall structure of the data. First, coinfection was not randomly distributed across the pediatric age range but concentrated on younger children. Second, among the etiologic categories examined, only viral coinfection showed a clear independent association with respiratory failure after adjustment. Third, biologic differences across groups were present, but they did not form a uniform “more coinfection = more inflammation” gradient; rather, they suggested distinct phenotypic profiles across etiologic classes. These findings refine the interpretation of pediatric MP coinfection beyond a purely descriptive characterization of the cohort.

The age effect was the dominant epidemiologic signal in the dataset. At the descriptive level, children with MP monoinfection were older than those with coinfection, with median ages of 11 years in the monoinfection group, 6.5 years in the viral coinfection group, and 5 years in the bacterial/mixed group, in line with other studies [5,17]. This pattern was reinforced by the multinomial model, in which each additional year of age was associated with lower relative risk of both viral coinfection (RRR 0.86, 95% CI 0.79–0.94, *p* < 0.001) and bacterial/mixed coinfection (RRR 0.79, 95% CI 0.72–0.87, *p* < 0.001), using MP monoinfection as the reference category.

The present analysis supports a simple interpretation: coinfection in pediatric MP pneumonia is primarily a phenomenon of younger children. This age structuring is clinically relevant because it provides the background against which severity should be interpreted. Younger children are exposed to a denser respiratory pathogen environment and, at the same time, have less mature airway and mucosal immune defenses [18,19]. In that setting, multiplex detection of more than one pathogen is not unexpected. However, the data do not support treating all coinfections as equivalent. The key result of the present analysis is not that “coinfection” in general worsens disease, but that the viral coinfection subgroup carries the clearest excess respiratory risk.

Bacterial/mixed coinfection with MP predominated (18.2%) in our study, followed by viral coinfection (17.7%). By contrast, Li Yuan et al.’s research found a lower rate of 9.1% for MP + bacteria and 11.6% for MP + viruses + bacteria, while viral coinfecting pathogens were dominant at 19.8% [8].

Regarding coinfecting pathogens in MP infections, *H. influenzae* and HRV predominated, consistent with previous studies [20,21]. However, Aosong Yu et al. found HAdV to be the most common pathogen involved in coinfection [8], while other studies reported *S. pneumoniae* [5,17], HBoV [5] and HPIV [22] as the leading etiologic agents in MP coinfection in the pediatric population. These differences may reflect variations in sample size, age distribution, local circulation of respiratory pathogens, testing strategy, and seasonality, and, since in the present cohort pathogen-level comparisons were not reliable due to most individual copathogens occurring in small numbers, the viral and bacterial/mixed groups should be read as broad co-detection categories rather than as pathogen-specific entities. This is a key point to consider, especially since adenovirus, influenza viruses, rhinovirus, *S. pneumoniae*, and *H. influenzae* are unlikely to have identical clinical effects; however, much larger prospective cohorts would be needed to separate these pathogen-specific contributions at acceptable levels of precision.

One additional point is that the viral and bacterial categories used in this analysis necessarily bring together pathogens with different biological behavior. For example, respiratory viruses such as adenovirus or influenza may produce more pronounced epithelial injury and inflammatory activation than other viruses, while bacterial copathogens such as Streptococcus pneumoniae may affect disease evolution through mechanisms related to invasion, inflammation, or secondary bacterial injury. Our cohort was not large enough to support reliable pathogen-level stratification, and further subdivision led to very small subgroups. For this reason, the associations should be interpreted at the level of broad etiologic classes, not as effects of individual pathogens. Larger prospective cohorts would be needed to separate these pathogen-specific contributions more clearly.

Respiratory failure was the most informative clinical outcome in the cohort. Its crude prevalence was 36% in MP monoinfections, 65% in MP + viral coinfection, and 31% in MP + bacterial/mixed coinfection, and this difference persisted after adjustment. In the logistic regression model, viral coinfection was associated with higher odds of respiratory failure relative to MP monoinfections (OR 3.37, 95% CI 1.49–7.94, *p* = 0.004), whereas bacterial/mixed coinfection was not (OR 0.85, 95% CI 0.35–2.03, *p* = 0.725). This is the central inferential result of the study. The data therefore support a more specific interpretation: the clearest adjusted respiratory severity signal is linked to viral coinfection rather than to coinfection considered as a single undifferentiated category. From a biological perspective, viral copathogens may contribute to airway injury and amplify local inflammatory responses beyond what would be expected from pathogen detection alone [23,24]. One possible explanation involves MP-related immune activation pathways linked to CARDS toxin, although the present study was not designed to evaluate these mechanisms directly. CARDS toxin has been shown to activate the NLRP3 inflammasome and promote release of proinflammatory cytokines, including IL-17 and IL-18 [25]. In this context, concomitant viral infection may enhance inflammatory signaling and epithelial damage, particularly in younger children whose airway and immune responses are still developing. Th17-associated inflammation and neutrophil recruitment have also been implicated in MP-related airway injury [25,26,27].

At the same time, our findings should be interpreted cautiously. The study was not designed to determine pathogen-specific mechanisms, and the broad viral grouping used for analysis remains a limitation. Nevertheless, the association between viral coinfection and respiratory failure was observed both descriptively and after multivariable adjustment, suggesting that the finding is unlikely to be explained solely by statistical artifact. At the same time, the modest ROC performance of the respiratory failure model indicates that etiologic grouping alone is insufficient for individual-level prediction. In practical terms, viral coinfection is an important severity marker at the group level, but not a stand-alone predictive instrument. That distinction matters clinically and addresses reviewers’ concern about overstating applicability. Other recent analyses have shown that MP pneumonia coinfection results in a more severe symptomatology and outcome [28,29,30,31]. For instance, MP pneumonia coinfected with HAdV was correlated with more severe clinical course (prolonged pyrexia, higher rate of dyspnea, extended length of stay and increased need for oxygen therapy), with longer duration of fever, massive consolidation on X-ray and more extrapulmonary complications [32,33]. Although the underlying mechanism has not been fully elucidated, a synergistic relationship has been postulated in which adenovirus induces marked epithelial destruction, facilitating pneumococcal invasion and exacerbating the *M. pneumoniae*-mediated immune response [32,34]. New findings regarding the potential mechanism were reported by Wenxiang Zhou et al. in their research, where they compared the features of the bronchoalveolar lavage fluid (BALF) microbiome composition between MP single infection and RMPP coinfected with HAdV. They found a higher β-diversity in the coinfection group, increasing intragroup difference [35].

A different study conducted by Choo et al. has revealed that MP patients with respiratory virus coinfection are prone to developing refractory MP pneumonia [18]. Moreover, it has been suggested that MP necrotizing pneumonia could be caused by severe bacterial or viral coinfections [33,36].

The length-of-stay analysis was less conclusive. Median LOS was similar across groups, and the negative binomial model did not provide strong evidence of a difference in hospitalization duration for either coinfection category relative to monoinfection, although the viral contrast trended upward (IRR 1.16, 95% CI 0.99–1.37, *p* = 0.069). Qing Song et al. [37] showed similar results regarding length of stay, while Agness Nicholaus Kanusya et al. [17] found a prolonged length of stay in MP patients coinfected with other pathogens. In another study conducted by Chih-Yung Chiu et al. [15], they observed that children with MP with *S. pneumoniae* coinfection required longer length of stay, and those associated with pleural effusion were transferred to the intensive care unit. Age showed a small positive association with LOS (IRR 1.01 per year, 95% CI 1.00–1.03, *p* = 0.049). Taken together, these results indicate a clearer etiologic signal for respiratory severity than for hospitalization duration.

That distinction improves the balance of the manuscript and avoids forcing the same narrative onto all outcomes. The laboratory data add depth to this picture, but they should not be overinterpreted. Several markers differed significantly across groups. WBC increased from a median of 8.61 × 10^9^/L in MP monoinfection to 10.38 in viral coinfection and 11.68 in bacterial/mixed coinfection (*p* < 0.001), while lymphocyte counts also increased across the same groups (*p* = 0.002). Hemoglobin and MCV were lower in the bacterial/mixed group than in monoinfection, and AST was higher in both coinfection groups than in MP alone. CRP, however, did not follow the same directional pattern and was lower in the bacterial/mixed group than in MP monoinfection, suggesting that CRP may reflect heterogeneous inflammatory kinetics across etiologic groups rather than a simple severity gradient. This is a counterintuitive patten that needs cautious interpretation, as it may reflect differences in timing of blood sampling, heterogeneity within the bacterial/mixed group, prior treatment before admission, or different inflammatory kinetics across pathogens, which makes CRP more useful as a descriptive marker than a severity indicator in this particular dataset. LDH was not significantly different across groups, with a similar finding being reported in another study [38]. Nevertheless, Wu X et al. reported in their study high levels of LDH in MP patients with viral coinfection [39].

These findings are more informative when read as evidence of biologic heterogeneity than as a single severity ladder. They suggest that etiologic categories may correspond to different inflammatory or systemic response profiles, rather than simply more-versus-less-severe manifestations of the same process. The etiologic groups should therefore not be considered biologically uniform. Within the viral coinfection group, individual viruses may differ in their effects on airway inflammation, epithelial integrity, and immune activation. The same applies to the bacterial/mixed group, which combines organisms with different pathogenic mechanisms. Consequently, the group-level differences observed in this study should be understood as average signals across heterogeneous subgroups, rather than as evidence of one shared biological pathway.

Among the coagulation-related markers, fibrinogen showed the clearest separation between groups, with lower values in the bacterial/mixed coinfection categories. This finding should not be read as evidence that fibrinogen can be used as an individual prognostic marker, but rather that it may reflect differences in inflammatory or coagulation responses across etiologic groups. Although fibrinogen abnormalities have been associated with severity in other infectious settings, our data does not establish its prognostic value in pediatric MP coinfection. Longitudinal studies are required to determine whether fibrinogen has practical clinical utility in this context. The present data do not support the use of fibrinogen as a validated prognostic marker or clinical decision tool. Rather, they suggest that the bacterial/mixed co-detection group may have a distinct biologic profile, a possibility that should be regarded as hypothesis-generating.

The study has several limitations. It was retrospective and single center, which limits generalizability, making the findings sensitive to local admission criteria, testing practice, and clinical management. Etiologic grouping was based on nasopharyngeal multiplex RT-PCR co-detection, which does not by itself establish causally relevant lower-respiratory infection for every detected copathogen. This issue is especially relevant in children, in whom asymptomatic carriage of respiratory viruses is frequent. Detection of viral nucleic acid in an upper respiratory tract sample does not necessarily prove active lower respiratory tract infection, nor does it establish that the detected virus contributed causally to the current episode. Therefore, the associations reported here are best interpreted as associations with co-detection patterns rather than direct pathogen-specific effects.

Biomarker availability was uneven across variables, making some laboratory comparisons less stable than the main cohort-wide analyses. In addition, the viral and bacterial/mixed categories were constructed as umbrella classes to improve statistical stability, but this necessarily pools pathogens with different biological behavior. The respiratory failure model also showed only modest discrimination, indicating that relevant determinants of severity remain incompletely captured. These considerations support interpreting the findings at the level of co-detection classes and adjusted associations rather than as pathogen-specific causal effects.

Taken together, these findings support a cautious interpretation, as MP coinfection in this cohort was mainly concentrated in younger children, and viral co-detection was the subgroup most clearly associated with respiratory failure, whereas the same pattern was not observed for bacterial or mixed co-detection, and the signal for length of stay was weaker, all of which form a strong argument against treating the results as a single clinical entity, and, considering the retrospective design, small subgroups, and use of upper-airway PCR, the findings should be considered hypothesis-generating rather than definitive.

Clinically, these results should mainly be read as a signal for closer observation, not as a basis for changing treatment. Children with MP and viral co-detection appeared more likely to develop respiratory failure, so this subgroup may deserve earlier recognition and careful monitoring during admission. However, our study did not compare therapeutic strategies, and treatment data were not analyzed in a standardized way. For that reason, we cannot say whether antibiotic choice, antiviral treatment, corticosteroids, or other supportive measures should differ between etiologic groups. This question will need prospective studies in which treatment exposure and clinical outcomes are collected in a more structured manner.

## 4. Materials and Methods

### 4.1. Study Design and Cohort Definition

This retrospective cohort study analyzed hospitalized pediatric patients with *Mycoplasma pneumoniae* infection at the Children’s Emergency Clinical Hospital “Sf. Ioan”, Galați, Romania, between April 2023 and November 2025. All children aged 0–17 years with PCR-confirmed *Mycoplasma pneumoniae* infection detected by multiplex respiratory panels during hospitalization were eligible for inclusion. The present analysis retained all eligible cases with available etiologic and clinical outcome data to preserve statistical stability for multivariable modeling. The final analytic cohort comprised 192 children, representing all available hospitalized cases meeting the diagnostic definition during the study period. Patients were classified according to a revised three-group etiologic framework: MP monoinfection, MP with viral coinfection, and MP with bacterial or mixed coinfection. This structure was selected to reduce subgroup fragmentation and to improve the stability of regression estimates. The study was approved by the Ethics Committee of the Children’s Emergency Clinical Hospital “Sf. Ioan” (approval number 25116, 18 November 2025).

### 4.2. Data Collection

Nasopharyngeal exudate was collected from each patient using synthetic fiber swabs with a plastic rod, which were introduced into 3 mL of universal transport medium (UTM) in sterile tubes. Samples were processed immediately or stored at −70 degrees until processing.

For the detection of MP, the Allplex™ Respiratory Panel Assays kit 4 (Seegene Inc., Seoul, Republic of Korea) was used, which allowed for simultaneous detection of bacteria such as *Bordetella pertussis*, *Bordetella parapertussis*, *Streptococcus pneumoniae*, *Haemophilus influenzae*, *Chlamydophila pneumoniae* and *Legionella pneumophila*. In addition, patients were also tested for 15 other respiratory viruses with Respiratory Panel Assays kits 1, 2, and 3 (Seegene Inc., Seoul, Republic of Korea): human rhinovirus A/B/C, human bocavirus 1/2/3/4, human enterovirus, human adenovirus, human metapneumovirus, human coronaviruses (229E, NL63, OC43), human parainfluenza 1/2/3/4, Influenza A/B, RSV and SARS-CoV-2. All included patients were tested with 4 multiplex RT-PCR respiratory panels strategy during hospitalization, and coinfections were identified when MP and at least one additional respiratory pathogen were detected in the same testing episode.

Nucleic acid extraction was performed with the Nimbus automatic extractor (Seegene Inc., Seoul, Republic of Korea) using the STARMag 96 × 4 universal kit (Seegene Inc., Seoul, Republic of Korea). The CFX96 amplifier (BIO-RAD, Hercules, CA, USA) was used for qRT-PCR testing.

### 4.3. Etiologic Grouping, Definitions and Clinical Outcomes

Patients were classified into three mutually exclusive etiologic groups based on multiplex RT-PCR detection patterns, with MP monoinfection defined as detection of *Mycoplasma pneumoniae* without any additional respiratory pathogen, MP + viral coinfection defined as MP detection together with at least one respiratory virus and no bacterial copathogen, and MP + bacterial/mixed coinfection was defined as MP detection together with at least one bacterial copathogen, with or without additional viral detection. The viral pathogens included human rhinovirus A/B/C, human bocavirus 1/2/3/4, human enterovirus, human adenovirus, human metapneumovirus, seasonal human coronaviruses, human parainfluenza viruses 1–4, influenza A/B, respiratory syncytial virus, and SARS-CoV-2. Bacterial pathogens included *Bordetella pertussis*, *Bordetella parapertussis*, *Streptococcus pneumoniae*, *Haemophilus influenzae*, *Chlamydophila pneumoniae*, and *Legionella pneumophila.* Clinical variables were extracted from the medical records, including ICU admission, oxygen therapy, pleural effusion, pulmonary consolidation, and respiratory failure, which was defined according to physician documentation and/or clinically significant respiratory compromise requiring oxygen therapy or respiratory support. Moreover, laboratory variables were recorded from the first available blood tests obtained within the initial hospital evaluation period after admission, and when repeated measurements were available, only the earliest values were retained for analysis.

Documented comorbidities and associated chronic conditions were identified from diagnostic and complication fields when explicitly reported in the medical records, and, because these data were retrospectively extracted from routine clinical documentation rather than collected using a standardized baseline comorbidity form, they were summarized descriptively and were not included as covariates in the adjusted regression models.

### 4.4. Statistical Analysis

All analyses were conducted in R using a prespecified workflow designed to distinguish descriptive comparisons from adjusted inference. Categorical variables were summarized as counts and percentages, and continuous variables as medians with interquartile ranges. Between-group descriptive comparisons across the revised three-level etiologic classification were performed using Fisher’s exact test for categorical variables and Kruskal–Wallis rank-sum testing for continuous variables.

The main adjusted analyses addressed three related questions. Respiratory failure was analyzed using multivariable logistic regression, with effect estimates reported as odds ratio (OR) and 95% confidence interval (CI), while length of stay was modeled using negative binomial regression to account for overdispersion, with results reported as incidence rate ratio (IRR) and 95% CI, and negative binomial regression was selected because length of stay was a count outcome with a skewed distribution. Predictors of etiologic group membership were evaluated using multinomial logistic regression with MP monoinfection as the reference category, and results were expressed as relative risk ratio (RRR) with 95% CI.

Hence, the etiologic framework used for inferential modeling comprised MP monoinfection, MP with viral coinfection, and MP with bacterial or mixed coinfection. This structure was selected to reduce subgroup fragmentation and improve estimate stability relative to the earlier four-group exploratory framework. Core demographic covariates were retained in adjusted models to improve clinical interpretability and reduce confounding by age structure, sex, and residence. Laboratory comparisons were treated as descriptive and exploratory. Model outputs were complemented by forest plots, marginal prediction plots, and receiver operating characteristic analysis for the respiratory failure model. All tests were two-sided, and inference was based primarily on effect sizes and 95% confidence intervals rather than on *p*-values alone.

### 4.5. Cohort Size Considerations

The full cohort of hospitalized children with PCR-confirmed *Mycoplasma pneumoniae* infection during the study period was retained for analysis (*n* = 192) to preserve subgroup stability and minimize variance inflation across etiologic strata. The final dataset comprised MP monoinfection (*n* = 123), MP + viral coinfection (*n* = 34), and MP + bacterial/mixed coinfection (*n* = 35).

Because the study was not prospectively powered, inferential stability was evaluated post hoc using a precision-based framework combining confidence interval reconstruction and approximate achieved power estimation. These analyses are presented as a methodological supplement, whereas effect sizes and confidence intervals remain the primary basis for inference.

For ratio measures (OR, IRR, and RRR), standard errors were reconstructed on the log scale from reported 95% confidence intervals, and approximate achieved power was derived from the corresponding Wald statistics under a two-sided α = 0.05. The principal etiologic contrast for respiratory failure, MP monoinfection versus MP + viral coinfection, showed a non-null effect with exclusion of unity (OR 3.37, 95% CI 1.49–7.94). The bacterial/mixed contrast was less precise and compatible with the null value (OR 0.85, 95% CI 0.35–2.03). Length-of-stay contrasts were weaker and less precise, whereas age effects in the multinomial model showed consistently narrow intervals, supporting stable estimation of the age structure of coinfection.

**Figure 8:**
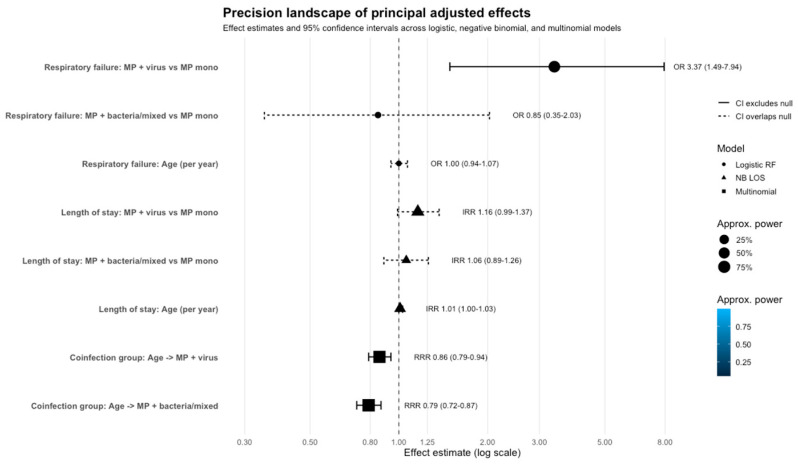
Precision landscape of principal adjusted effects. Caption: Adjusted effect estimates (OR, IRR, RRR) and 95% confidence intervals from the logistic respiratory—failure model, negative binomial length–of–stay model, and multinomial etiologic—group model. Marker size and color encode approximate achieved power derived from reconstructed log–scale standard errors; larger and warmer-colored markers indicate higher approximate achieved power, while the dashed vertical line denotes the null value (ratio = 1). Viral coinfection showed the largest adjusted respiratory failure effect (OR 3.37, 95% CI 1.49–7.94), whereas the bacterial/mixed contrast was null–compatible (OR 0.85, 95% CI 0.35–2.03). Age effects in the multinomial model showed narrow intervals, reflecting strong age structuring of coinfection.

The distribution of adjusted effect estimates, confidence intervals, and approximate achieved power across principal regression contrasts is summarized in Figure 8.

**Figure 9:**
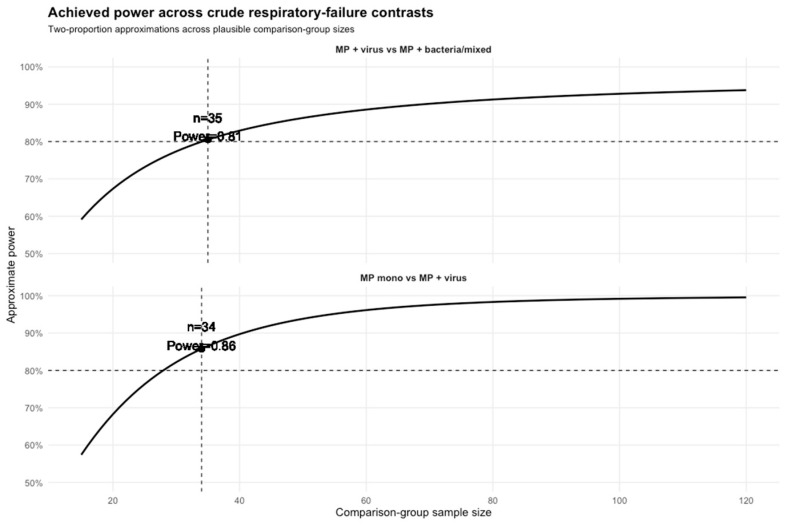
Achieved power across crude respiratory failure contrasts. Caption: Two-proportion approximations relating comparison-group sample size to statistical power for the observed respiratory failure differences. Horizontal dashed lines indicate the 80% power threshold; vertical dashed lines denote observed subgroup sizes. Observed viral subgroup size (*n* = 34) corresponds to achieved power ≈0.86 for MP monoinfection vs. MP + viral coinfection, while *n* = 35 corresponds to ≈ 0.81 for MP + viral vs. MP + bacterial/mixed coinfection. Power estimates are shown for contextualization and do not replace confidence-interval-based inference. The annotation labels identify the observed subgroup size and corresponding approximate achieved power for each crude contrast.

To contextualize cohort size relative to the primary clinical endpoint, achieved power curves were generated for the principal crude respiratory failure contrasts using two-proportion approximations. At the observed subgroup sizes, approximate achieved power was 0.86 for MP monoinfection vs. MP + viral coinfection (*n* = 34) and 0.81 for MP + viral vs. MP + bacterial/mixed coinfection (*n* = 35) (Figure 9).

## 5. Conclusions

In this single-center retrospective cohort of hospitalized children with PCR-confirmed MP infection, coinfection was observed more often in younger patients. Viral co-detection showed the clearest association with respiratory failure, whereas bacterial/mixed co-detection was not associated with increased respiratory failure in the adjusted model. The association with length of stay was weaker and less precise. Laboratory differences, including lower fibrinogen values in the bacterial/mixed group and the non-linear CRP pattern, suggest possible heterogeneity across etiologic groups, although these findings remain exploratory and should not be interpreted as evidence of validated prognostic biomarkers. Taken together, the findings suggest that viral and bacterial/mixed co-detection patterns may differ clinically in pediatric MP infection; however, the retrospective design, modest subgroup size, and upper-airway PCR-based pathogen detection require cautious interpretation, and the results should be considered hypothesis-generating rather than definitive.

Clinically, viral co-detection may identify a subgroup of children at higher risk for respiratory compromise who could benefit from closer monitoring during hospitalization. However, treatment implications remain uncertain because therapeutic strategies were not analyzed in a standardized manner. Larger prospective studies with standardized sampling, comorbidity assessment, treatment data, and pathogen-level analyses are perhaps needed to determine whether these observed patterns have reproducible clinical significance.

## Figures and Tables

**Table 1:** Clinical and demographic characteristics by etiologic group.

Characteristic	Overall *n* = 192 ^1^	MP *n* = 123 ^1^	MP + Virus *n* = 34 ^1^	MP + Bacteria ± Virus *n* = 35 ^1^	*p* Value ^2^
Sex					0.3
Male	114 (59%)	71 (58%)	24 (71%)	19 (54%)	
Female	78 (41%)	52 (42%)	10 (29%)	16 (46%)	
Age_group (years)					<0.001
<1	16 (8.3%)	2 (1.6%)	8 (24%)	6 (17%)	
1–3	22 (11%)	9 (7.3%)	2 (5.9%)	11 (31%)	
4–6	32 (17%)	17 (14%)	7 (21%)	8 (23%)	
7–17	122 (64%)	95 (77%)	17 (50%)	10 (29%)	
Age (years)	9.00 (5.00, 13.00)	11.00 (7.00, 14.00)	6.50 (1.00, 11.00)	5.00 (1.00, 7.00)	<0.001
Length of stay (days)	7.00 (5.00, 8.00)	7.00 (5.00, 8.00)	7.00 (6.00, 9.00)	6.00 (4.00, 9.00)	0.2
ICU admission	33 (17%)	20 (16%)	7 (21%)	6 (17%)	0.8
Oxygen therapy	8 (4.2%)	5 (4.1%)	2 (5.9%)	1 (2.9%)	0.8
Fever	136 (71%)	91 (74%)	23 (70%)	22 (63%)	0.4
Rhinorrhea	26 (14%)	8 (6.5%)	9 (26%)	9 (26%)	<0.001
Cough > 7 days	26 (17%)	15 (15%)	4 (16%)	7 (28%)	0.3
Dyspnea	37 (19%)	25 (20%)	6 (18%)	6 (17%)	0.9
Vomiting	22 (11%)	10 (8.1%)	6 (18%)	6 (17%)	0.11
Wheezing	10 (5.2%)	4 (3.3%)	4 (12%)	2 (5.7%)	0.11
SpO_2_ < 92%	4 (3.8%)	4 (5.9%)	0 (0%)	0 (0%)	0.6
Rales	138 (72%)	91 (74%)	25 (74%)	22 (63%)	0.4
Respiratory failure	77 (40%)	44 (36%)	22 (65%)	11 (31%)	0.005
Pleural effusion	19 (11%)	15 (14%)	1 (3.3%)	3 (11%)	0.3
Lung consolidation	77 (46%)	55 (50%)	12 (40%)	10 (36%)	0.3
Documented comorbidities	36(18.8%)	24(19.5%)	3(8.8%)	9 (25.7%)	0.170
Asthma	10 (5.2%)	6 (4.9%)	1 (2.9%)	3 (8.6%)	0.630
Obesity	8 (4.2%)	7 (5.7%)	0 (0%)	1 (2.9%)	0.513
Poor nutritional status	12 (6.2%)	8 (6.5%)	1 (2.9%)	3 (8.6%)	0.625
Neurologic disease	4 (2.1%)	3 (2.4%)	1 (2.9%)	0 (0%)	0.807
Congenital heart disease	1 (0.5%)	1 (0.8%)	0 (0%)	0 (0%)	1.000
Adenoid hypertrophy	4 (2.1%)	2 (1.6%)	0 (0%)	2 (5.7%)	0.291

Caption: Demographic variables, hospitalization parameters, and presenting clinical features are shown across the revised three-group etiologic framework: MP monoinfection, MP + viral coinfection, and MP + bacterial/mixed coinfection. Continuous variables are presented as median (IQR) and categorical variables as *n* (%). The *p* values were derived from Kruskal–Wallis rank-sum testing for continuous variables and Fisher’s exact test for categorical variables. ICU, intensive care unit; LOS, length of stay. Comorbidities were extracted when explicitly documented in diagnostic or complication fields and were summarized descriptively. ^1^ *n* (%); median (Q1, Q3). ^2^ Fisher’s exact test; Kruskal–Wallis rank sum test.

**Table 2:** Hematological and biochemical profile by etiologic group.

Characteristic	Overall *n* = 192 ^1^	MP *n* = 123 ^1^	MP + Virus *n* = 34 ^1^	MP + Bacteria ± Virus *n* = 35 ^1^	*p* Value ^2^
WBC (×10^9^/L)	9.17 (7.54, 11.84)	8.61 (7.01, 10.41)	10.38 (7.78, 13.26)	11.68 (9.01, 14.58)	<0.001
Neutrophils (×10^3^/µL)	5.69 (4.25, 7.84)	5.50 (4.34, 6.89)	5.71 (3.67, 8.29)	6.68 (4.22, 10.80)	0.3
Lymphocytes (×10^3^/µL)	2.07 (1.42, 3.23)	1.77 (1.25, 2.76)	2.18 (1.56, 3.28)	2.98 (1.84, 4.07)	0.002
Hb (g/dL)	12.90 (11.92, 13.65)	13.10 (12.25, 13.71)	12.87 (11.60, 13.66)	12.37 (11.23, 13.10)	0.002
MCV (fL)	80.30 (76.90, 83.10)	80.80 (77.50, 83.80)	80.50 (77.40, 81.70)	77.50 (72.50, 81.50)	0.017
CRP (mg/dL)	2.05 (0.70, 4.61)	2.66 (0.83, 4.82)	1.96 (0.61, 3.92)	0.91 (0.66, 2.00)	0.039
AST (U/L)	30.00 (25.00, 37.00)	28.00 (24.00, 35.00)	34.00 (27.00, 40.00)	34.00 (28.00, 38.00)	0.002
LDH (UI/L)	260.00 (194.00, 298.00)	258.00 (186.00, 289.00)	277.00 (214.00, 331.00)	266.50 (204.00, 305.00)	0.15

Caption: Hematologic and inflammatory markers are presented across the three etiologic groups. Continuous variables are summarized as median (IQR). The *p* values were obtained using Kruskal–Wallis rank-sum testing. WBC, white blood cell count; MCV, mean corpuscular volume; CRP, C-reactive protein; AST, aspartate aminotransferase; LDH, lactate dehydrogenase. ^1^ Median (Q1, Q3). ^2^ Kruskal–Wallis rank sum test.

**Table 3:** Coagulation profile by etiologic group.

Characteristic	Overall *n* = 192 ^1^	MP *n* = 123 ^1^	MP + Virus *n* = 34 ^1^	MP + Bacteria ± Virus *n* = 35 ^1^	*p* Value ^2^
Fibrinogen (mg/dL)	409.00 (327.50, 496.00)	439.00 (353.00, 497.50)	372.00 (315.50, 505.50)	284.00 (223.50, 321.00)	0.004
D-dimer (µg/mL)	0.66 (0.44, 1.16)	0.73 (0.42, 1.64)	0.63 (0.54, 1.01)	0.53 (0.36, 0.93)	0.7

Caption: Coagulation-related biomarkers across MP monoinfection, MP + viral coinfection, and MP + bacterial/mixed coinfection. Values are shown as median (IQR). The *p* values were calculated using Kruskal–Wallis rank-sum testing. The most pronounced group difference was observed for fibrinogen, whereas D-dimer showed no significant between-group variation. ^1^ Median (Q1, Q3). ^2^ Kruskal–Wallis rank sum test.

**Table 4:** Logistic regression model for respiratory failure.

Logistic Regression—Respiratory Failure (Adjusted OR)			
Predictor	Effect	95% CI	*p*
(Intercept)	0.58	0.25–1.36	0.218
MP + virus	3.37	1.49–7.94	0.004
MP + bacteria ± virus	0.85	0.35–2.03	0.725
Age (years)	1.00	0.94–1.07	0.981
Sex1	1.07	0.58–1.98	0.821
Residence2	0.79	0.41–1.48	0.460

Caption: Adjusted odds ratios for respiratory failure according to etiologic group and demographic covariates. MP monoinfection was used as the reference category for etiologic comparisons. Results are presented as OR with 95% confidence intervals and *p* values. OR, odds ratio; CI, confidence interval.

**Table 5:** Negative binomial regression model for length of stay.

Negative Binomial—Length of Stay (Adjusted IRR)			
Predictor	Effect	95% CI	*p*
(Intercept)	6.01	5.04–7.15	<0.001
MP + virus	1.16	0.99–1.37	0.069
MP + bacteria ± virus	1.06	0.89–1.26	0.525
Age (years)	1.01	1.00–1.03	0.049
Sex1	1.02	0.90–1.15	0.742
Residence2	1.02	0.90–1.16	0.760

Caption: Adjusted incidence rate ratios for length of stay according to etiologic group and demographic covariates. MP monoinfection was used as the reference category for etiologic contrasts. Results are presented as IRR with 95% confidence intervals and *p* values. IRR, incidence rate ratio; CI, confidence interval.

**Table 6:** Multinomial regression model for predictors of etiologic group membership.

Multinomial Logistic Regression—Coinfection Group (RRR vs. MP Mono)				
Outcome Level (vs. Ref)	Predictor	Effect	95% CI	*p*
MP + virus	Intercept	1.21	0.49–3.00	0.686
MP + virus	Age (per year)	0.86	0.79–0.94	<0.001
MP + virus	Sex (1 vs. 0)	0.50	0.21–1.18	0.114
MP + virus	Residence (2 vs. 1)	1.14	0.49–2.65	0.759
MP + bacteria ± virus	Intercept	1.34	0.52–3.45	0.541
MP + bacteria ± virus	Age (per year)	0.79	0.72–0.87	<0.001
MP + bacteria ± virus	Sex (1 vs. 0)	0.94	0.40–2.20	0.891
MP + bacteria ± virus	Residence (2 vs. 1)	1.87	0.80–4.36	0.150

Caption: Relative risk ratios for predictors belonging to the MP + viral coinfection group or the MP + bacterial/mixed coinfection group, using MP monoinfection as the reference category. Results are presented as RRR with 95% confidence intervals and *p* values. RRR, relative risk ratio; CI, confidence interval.

## Data Availability

The raw data supporting the conclusions of this article will be made available by the authors on request.

## Abbreviations

The following abbreviations are used in this manuscript:

ICU	Intensive care unit
MP	*Mycoplasma pneumoniae*
HI	*Haemophilus influenzae*
SP	*Streptococcus pneumoniae*
HRV	Human rhinovirus
HPIV	Human parainfluenza virus
HCoV	Human coronavirus
HAdV	Human adenovirus
HBoV	Human bocavirus
RMPP	Refractory Mycoplasma pneumoniae pneumonia
RSV	Respiratory syncytial virus
LDH	Lactate dehydrogenase
AST	Aspartate aminotransferase
ALT	Alanine aminotransferase
CRP	C-reactive protein
MCV	Mean corpuscular volume
ESR	Erythrocyte sedimentation rate
WBC	White blood cells
CARDS	Community-acquired respiratory distress syndrome toxin
BALF	Bronchoalveolar lavage fluid
aOR	Adjusted odds ratio
CI	Confidence interval
LOS	Length of stay
IL	Interleukin
Ig	Immunoglobulin
RF	Respiratory failure
SD	Standard deviation
IQR	Interquartile range
CAP	Community-acquired pneumonia
ROS	Reactive oxygen species
CDC	Centers for Disease Control and Prevention
SpO_2_	Oxygen saturation level measured by a pulse oximeter
UTM	Universal transport medium
qRT-PCR	Quantitative reverse transcription polymerase chain reaction
IRR	Incidence rate ratio
RRR	Relative risk ratio
